# Changes in gross motor function in children with cerebral palsy following repeated intensive rehabilitation periods: a longitudinal study

**DOI:** 10.3389/fped.2025.1636955

**Published:** 2025-11-18

**Authors:** Viktoria Opheim, Camilla Skagen, Kine Melfald Tveten, Stian Lydersen, Gunfrid Vinje Størvold, Rannei Sæther

**Affiliations:** 1 Department of Physiotherapy Services for Children and Youth, Interdisciplinary Resource Team, Tromsø, Norway; 2 Children’s Physiotherapy Center, Bergen, Norway; 3 Department of Health and Functioning, Faculty of Health and Social Science, Western Norway University of Applied Sciences, Bergen, Norway; 4 Regional Centre for Child and Youth Mental Health and Child Welfare (RKBU), Department of Mental Health, Faculty of Medicine and Health Sciences, Norwegian University of Science and Technology (NTNU), Trondheim, Norway; 5 Department of Habilitation, Levanger Hospital, Nord-Trøndelag Hospital Trust, Levanger, Norway; 6 Regional Centre for Habilitation, Department of Mental Health, Faculty of Medicine and Health Sciences, Norwegian University of Science and Technology (NTNU), Trondheim, Norway; 7 Department of Rehabilitation Science and Health Technology, Faculty of Health Sciences, Oslo Metropolitan University, Oslo, Norway

**Keywords:** cerebral palsy, GMFM-66, intensive rehabilitation, gross motor function, group training, longitudinal study

## Abstract

**Purpose:**

To evaluate changes in gross motor function in children with cerebral palsy (CP), following multiple intensive rehabilitation periods.

**Materials and methods:**

In this retrospective longitudinal study, we assessed 46 children with CP, aged 2–12 years, Gross Motor Function Classification System (GMFCS) levels I–V. Participants underwent 2–12 intensive rehabilitation periods, incorporating goal-directed group activities. Long-term change in gross motor function was analyzed using a linear mixed model based on reference percentiles for the Gross Motor Function Measure-66 (GMFM-66). Comparisons were made between GMFCS levels and age groups.

**Results:**

GMFM-66 scores increased during each intensive rehabilitation period, with significant improvements in period 1–4. The number of participants decreased as the number of periods increased. GMFM-66 percentiles increased by 10.9 (*p* < 0.001) per period, with an additive gain of 2.4 (*p* = 0.124) GMFM-66 percentiles per added intensive rehabilitation period. A decrease in percentiles was observed between periods. Gross motor function enhanced across all GMFCS levels, particularly levels I and V. Children under 6 years showed greater improvements than older children.

**Conclusions:**

Intensive rehabilitation may be associated with improved gross motor function in children with CP, irrespective of GMFCS levels. Repeated periods appear especially beneficial in early childhood, supporting their strategic use in long-term rehabilitation.

## Introduction

Cerebral palsy (CP) is the most common childhood disability (1) and is traditionally defined as “a group of permanent disorders of the development of movement and posture, causing activity limitation, that are attributed to non-progressive disturbances that occurred in the developing fetal or infant brain” (2). More recently, CP has been conceptualized as an early-onset, lifelong neurodevelopmental condition characterized by limitations in activity due to impaired development of movement and posture, resulting from non-degenerative brain dysplasia or injury in early life, and frequently accompanied by additional impairments across multiple domains (3). CP is associated with a wide range of activity and participation limitations (4, 5), with severity of impaired gross motor function classified by the five-level Gross Motor Function Classification System (GMFCS) (6). Motor impairments are often accompanied by secondary musculoskeletal conditions and disturbances in sensory-, cognitive-, communicative- and behavioral functions (2), which consequently may require a multidisciplinary approach throughout life. As activity limitations in gross motor function are a core symptom in CP (7), identifying strategies to improve gross motor skills is important for enhancing the quality of life (8, 9). Improving gross motor function may positively influence a child's ability to navigate their environment, engage in play, and interact in educational and community activities (8, 10).

The International Classification of Functioning, Disability, and Health (ICF) (11), along with system theories on motor learning (12, 13), consider gross motor progress a result of interactions between multiple factors, including child characteristics, environmental factors and interventions (14). In recent years, the CP treatment evidence base has expanded rapidly, providing information about more effective interventions (15). High-intensity, task-oriented training that incorporates real-life, goal-directed activities and motor learning principles (e.g., intensity, specificity, salience) has shown positive effects on gross motor function in children with CP (10, 15–18). Thus suggesting that training must be sufficiently intense to stimulate experience-dependent neural plasticity (12).

Motor function serves as the most common outcome parameter in research involving individuals with CP. This is frequently evaluated using the Gross Motor Function Measure-66 (GMFM-66) due to its robust psychometric properties (19, 20). Particular attention is directed towards intensive treatments, indicating that high-intensity training is effective in improving short-term gross motor progress in children with CP (21–23). In Norway, intensive rehabilitation programs function as a supplemental approach to local habilitation services, with the primary objective of amplifying activity levels and participation in daily life (24, 25).

Generally, children classified as GMFCS levels I–II exhibit more pronounced improvements in gross motor functions after intensive rehabilitation compared to those classified in levels III–V (26, 27). Similarly, the most significant gross motor progress primarily occurs early in life and in younger children (21, 27, 28). However, it should be noted that children with CP typically show an increase in gross motor function up to the age of 6–7 years under standard care (13, 26). Long-term changes in gross motor function have only been explored in a limited number of studies (14, 26, 29), which have reported cumulative effects on gross motor function following multiple periods of intensive rehabilitation. Nevertheless, the intensity and longevity of the intensive periods vary significantly in the studies, resulting in insufficient evidence to the role the amount and intensity of intensive rehabilitation plays in changes in gross motor function beyond natural progression.

Longitudinal monitoring of gross motor function allows for the identification of developmental trajectories and facilitating early intervention strategies (30). Assessing the long-term implications intensive rehabilitation has on changes in gross motor function may provide important insights for healthcare professionals in optimizing treatment plans for children with CP. Therefore, the primary aim of this longitudinal study was to evaluate which changes in gross motor function could be observed in children with CP after repeated periods of intensive rehabilitation, assessed using the GMFM-66. The secondary aim was to investigate whether changes in gross motor function differ between GMFCS levels, and if children exhibit different patterns of change dependent on age groups.

## Materials and methods

### Design

This study uses a retrospective longitudinal research design, incorporating repeated data from the Children's Physiotherapy Center (CPC) in Bergen, Norway, over 6 years, from 2017 to 2023. The sample of children was obtained from the data materials of CPC's project “Intensity matters” (31). Participants were recruited from different regions of Norway. Inclusion criteria of the original sample were children diagnosed with significantly reduced motor function due to congenital or early acquired brain damage, from the age of 6 months to 16 years. Exclusion criteria were parents with poor understanding of Norwegian or English.

### Participants

Forty-six children aged between 2 and 12 years, diagnosed with CP across all GMFCS severity levels, were included in this study. The selection aimed to include children with CP across all severity levels, and to use gross motor reference percentiles, developed for children with CP, as the primary dependent variable for evaluating longitudinal changes.

### Intervention

The multidisciplinary intensive rehabilitation program at the CPC was carried out by a team of physiotherapists, occupational therapists, sports educators, and pediatricians. The program consisted of two three-week periods over a year, incorporating group training sessions Monday through Thursday, each lasting 5 h (31). Training groups, composed of 3–6 children and their parents, were tailored to align participants in terms of age, cognitive and functional ability. Before the intervention, individualized goals and plans were developed in collaboration with each child, their parent or guardian, and the therapist. Activities were designed to promote mastery, function, enjoyment of movement, development, and independence, with a focus on gross motor skills and goal-directed activities relevant to daily life, such as riding a bike, walking outdoors and climbing stairs (32). Tasks were a combination of structured group activities and individually tailored, goal-directed tasks.

The parents were active in the training and received close follow-up from the therapists (31). The parents learned through “hands on” training how they could continue training, stimulation, and facilitation at home. Contact was established between the CPC and the health service in the child's municipality to exchange knowledge about the participants and their goals. Between intensive rehabilitation periods, children received follow-up from local physiotherapists, focusing on set goals. However, the amount or content of local follow-up is not specified.

Children were initially offered two intensive rehabilitation periods. Eligibility for subsequent periods was determined by regional health authorities and, upon new referral, granted in sets of two. Recruitment occurred continuously from 2017 to 2023, resulting in variation in the total number of periods completed. Those enrolling earlier in the study could complete more intensive rehabilitation periods than those enrolled later. Consequently, the number of periods per child depended on enrollment timing and the study's data collection period. Occasionally, periods were postponed due to illness, medical treatment, or family circumstances, leading to long intervals of 2–3 years before resuming participation; however, such instances were rare.

### Outcome measures

The GMFM-66 was used to monitor changes in gross motor function in children with CP over time, due to its strong psychometric properties (19, 20, 33–35). The GMFM-66 assesses performance across five dimensions of gross motor activities, with a four-point scoring scale based on the extent of skill mastery rather than the quality of performance (36). The scores are calculated on a 0–100 interval scale, with children expected to start near 0 as newborns and acquire skills rapidly in early years, slowing as they reach their potential (34). The minimum clinically important difference (MCID) in change score has been studied, with a score of 1.58 indicating general improvement and a score of 3.71 indicating a large improvement (19).

In 2002, Rosenbaum and colleagues (7) published five motor development curves, corresponding to the GMFCS levels. These curves illustrated changes in GMFM-66 scores across severity levels, indicating both development pace and an anticipated ceiling for functional capabilities (7). The curves typically show rapid progression to peak GMFM-66 scores at younger ages, exhibiting a leveling of scores in levels I and II, or a decline followed by leveling for level III–V (13).

To enable more nuanced evaluations, Hanna et al. (28, 37) developed reference percentiles, providing an appropriate normative interpretation of GMFM-66 scores by age within each GMFCS level. These percentiles account for natural maturation and development, as they are based on a longitudinal sample of children with CP, reflecting typical age-related changes in motor function (28). Despite large variability (28), children with CP are generally expected to follow their GMFM-66 percentile over time (14), thus a positive change in the GMFM-66 percentile may indicate better motor development beyond the expected curve. Hanna et al. (28) reported mean percentile changes of 3.0, −0.8, 3.3, 2.5, and 3.6 for GMFCS levels I–V, respectively. Furthermore, a second assessment indicated about a 50% chance of being within ±10.5, 10.5, 8.4, 8.0, and 8.9 percentiles for each GMFCS level. In this study, GMFM-66 percentiles were used as the primary outcome measure, due to their ability to account for age-related expectations within each GMFCS level.

### Method of data collection

Children were assessed using the GMFM-66 on the first and last day of the program, and assessments were conducted at the CPC. The assessors were experienced physiotherapists familiar with the GMFM-66 measure. In most cases the same assessor was involved in both administering the pre- and post GMFM-66 testing and contributing to the group intervention as part of a multidisciplinary team.

### Statistical analysis

Statistical analyses were performed in IBM SPSS Statistics (Version 29) and Microsoft Excel. Descriptive statistics are presented as count (%) for categorical variables, and as mean (SD, min-max) for continuous variables. The Wilcoxon Signed Rank Test was applied to analyze changes throughout each intensive period.

The association between repeated periods of intensive rehabilitation and changes in gross motor function was analyzed utilizing a linear mixed model, with GMFM-66 percentiles as the primary dependent variable. The covariates were post-test (vs. pre-test), and the number of intensive rehabilitation periods. We included child as a random effect, with random intercept and random slope for the number of intensive rehabilitation periods. GMFM-66 scores were converted to GMFM-66 percentiles and subsequently to *z*-scores. A *z*-score equals the number of standard deviations from the mean. To better interpret the clinical implications of the results, outcomes using both percentiles and *z*-scores as dependent variables were reported. Participants with missing data at one or more time points are included in the linear mixed models with their available data. Results from linear mixed model analyses are unbiased if data are missing at random, while a complete case analysis including only participants with complete data would be unbiased only if data are missing completely at random, and also have less power (38).

A visual inspection of box plots revealed similar patterns in both models, whether using *z*-scores or GMFM-66 percentiles as the dependent variable. Consequently, due to its clinical interpretability, we present results for the GMFM-66 percentiles as the primary dependent variable.

To provide a more accurate representation of the intervention's impact on gross motor function according to the level of impairment and age progress, each GMFCS level and children under and over 6 years (at baseline) were analyzed separately. We conducted additional analyses that incorporated GMFCS or age group and their interactions with posttest and intensive rehabilitation periods. The selection of an age division under and over 6 years is based on the understanding that the most significant gross motor progress primarily occurs in early childhood and in younger children (13). Similarly, children typically show a plateauing in their GMFM-scores at that age (28).

To provide some protection against false positive findings due to multiple tests, we regard two-sided *p*-values under 0.01 to represent statistical significance.

### Ethical considerations

This study utilizes previously collected data, where the ethical approval was obtained from the Regional Committee for Medical and Health Research Ethics (2018/1781/REK vest) and by the Norwegian Social Science Data Service (project no. 898475). No protocol has been registered in ClinicalTrials, as the study is based on established practice. Parents or legal guardians signed informed consent letters before enrollment. If parents did not consent to participation, it did not impact their child's rehabilitation services.

## Results

### Demographical data and intervention periods

Demographic statistics are shown in Table 1. The age at the start of the intervention ranged from 24 to 131 months, with a mean of 61.8 months. All GMFCS levels were represented, but levels I, III and IV were most common. The majority had spastic bilateral CP. There were slightly more boys participating.

**Table 1:** Demographics of participants (*N* = 46).

Variable	*n*	%
Age at baseline^a^, in months; mean (SD), min-max.	61.8 (30.5), 24–131	
Sex		
Female	21	45.7
Male	25	54.3
GMFCS level		
I	10	21.7
II	5	10.9
III	10	21.7
IV	14	30.4
V	7	15.2
CP subtypes		
Spastic unilateral	10	21.7
Spastic bilateral	29	63.0
Ataxic	2	4.3
Dyskinetic	4	8.7
Unclassified	1	2.2

Categorical variables are presented as count (%), and continuous variables as mean (SD, min-max). GMFCS, gross motor function classification system.

a First intervention period.

**Figure 1:**
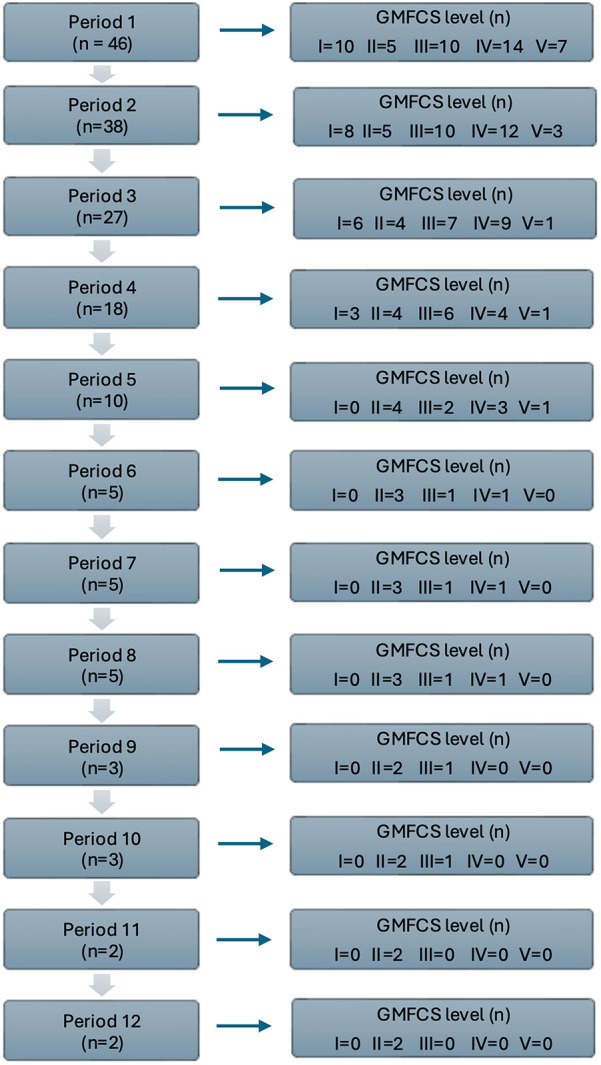
Flow chart of participant attrition across intensive rehabilitation periods; total and by GMFCS levels.

All participating children received at least one period with intensive rehabilitation, with GMFM-66 assessments pre- and post-intervention. While the program was initially planned to consist of two three-week periods over the course of a year, the intervals between these periods varied. Intervals between intensive rehabilitation periods ranged from 3 to 38 months, with a median (IQR) of 7.0 (5.0–8.0) months and a mean (SD) of 7.4 (4.4) months. The total distribution of intervals between intensive rehabilitation periods was calculated among participants who completed two or more periods, allowing for comparison of intervals between sessions. The number of participating children decreased as the number of intensive rehabilitation periods increased (Figure 1). Children with GMFCS level II comprised the largest group that underwent intensive rehabilitation for five or more periods.

### Long term change in gross motor function

Analysis of pre and post GMFM-66 scores, using the Wilcoxon signed rank test, demonstrated an increase in post-scores during all the intensive rehabilitation periods. The improvements were statistically significant for periods 1–4 (Table 2). For the majority of the intensive periods, changes exceeded the estimated MCID score (19), indicating a clinical meaningful progress. For the remaining periods, the number of participants decreased and the changes from pre- to post-intervention were not statistically significant (*p* ≥ 0.01).

**Table 2:** Comparison of median (min-max) GMFM-66 raw scores pre- and post-intervention for each intensive rehabilitation period.

Period	*n*	Pre-test median (min, max)	*n*	Post-test median (min, max)	Change score	*p*
1	46	47.2 (14.8, 96.0)	44	50.1 (21.3, 100)	2.9^b^	<.001*
2	38	48.5 (8.7, 81.9)	38	51.0 (10.3, 100)	2.5^b^	<.001*
3	27	56.9 (19.7, 100)	27	60.6 (26.0, 100)	3.7^b^	<.001*
4	18	60.8 (24.0, 87.9)	17	64.8 (26.6, 90.6)	4.0^b^	<.001*
5	10	64.4 (24.0, 82.4)	9	67.8 (40.7, 88.0)	3.4^b^	.012
6	5	68.5 (43.6, 70.8)	5	69.2 (43.4, 81.9)	0.7	.080
7	5	69.2 (42.2, 76.0)	5	69.6 (45.3, 81.9)	0.4	.043
8	5	68.0 (44.1, 79.9)	5	69.6 (49.6, 82.9)	1.6^b^	.109
9	3	69.2 (53.8, 80.9)	3	72.1 (55.1, 88.0)	2.9^b^	.109
10	3	74.2 (52.1, 76.8)	3	77.5 (53.8, 82.23)	3.3^b^	.109
11	2	80.6 (76.0, 85.2)	2	82.6 (79.9, 85.2)	2.0^b^	.317
12	2	81.4 (80.9, 81.9)	2	85.8 (81.9, 89.7)	4.4^b^	.180
13	1^a^	52.1	1^a^	54.7	2.6^b^	

a The *p*-value cannot be computed because there is only one observation.

b Change in GMFM-66 scores above the minimum clinically important difference (MCID).

* *p* < .001, statistically significant.

To investigate long-term changes in GMFM-66 percentiles after several periods of intensive rehabilitation, a linear mixed model was used (Table 3). Each period of intensive rehabilitation resulted in an estimated mean change of 10.9 (95% CI: 8.5–13.3. *p* < 0.001) in GMFM-66 percentiles, surpassing the expected average change in percentile score (28). An additive gain of multiple periods showed a mean increase of 2.4 GMFM-66 percentiles per added intensive rehabilitation period. However, this change was not statistically significant (*p* = 0.124).

**Table 3:** Estimated mean change (95% CI) in GMFM-66 percentiles^a^ according to each intensive rehabilitation period.

Independent variables	Estimate	95% CI	*p*
Intercept	53.0	45.4 to 60.7	<.001
Post test	10.9	8.5 to 13.3	<.001
Intensive rehabilitation periods	2.4	−0.7 to 5.6	.124

Post-test: change according to each intensive rehabilitation period, Intensive rehabilitation periods: the additive gain of multiple periods.

a Dependent variable.

**Figure 2:**
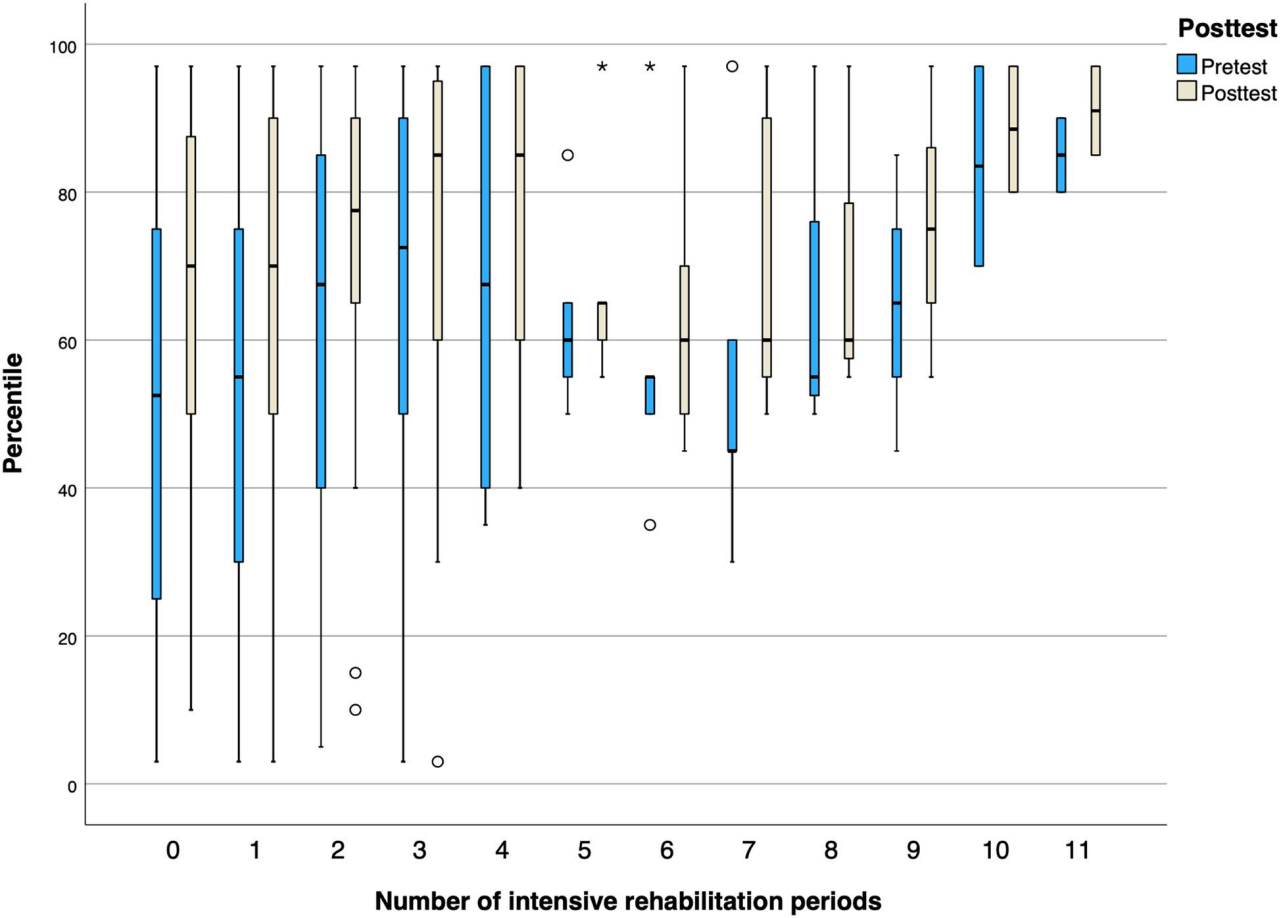
Box plot illustrating GMFM-66 percentile scores (0–100) pre- and post-test across intensive rehabilitation periods, highlighting changes over time. Note that no change in percentiles means progression as expected according to age and GMFCS level. A positive change in percentiles means an enhanced gross motor progress above what is expected according to age and GMFCS level.

An inspection of pre- and post-test GMFM-66 percentiles (Figure 2) showed a positive trend, with the largest improvements during the first five periods. A decrease in percentiles was observed after each rehabilitation period, but subsequent periods typically started, on average, at a higher level. A decrease in GMFM-66 percentiles during periods 5 and 6 may partly be due to the remaining participants after period 4 having lower scores (Figure 2).

### Comparison of changes in gross motor function across GMFCS levels

Comparisons revealed that intensive rehabilitation improved gross motor development across all GMFCS levels (*p* values ≤ 0.001). The greatest improvements were seen in children classified within GMFCS level I and V, with percentile changes of 18.7 (95% CI: 9.2–28.2) and 16.4 (95% CI: 7.7–25.1) respectively (Table 4).

**Table 4:** Estimated mean change (95% CI) in GMFM-66 reference percentiles^a^ according to each intensive rehabilitation period across GMFCS levels.

GMFCS	Independent variables	Estimate	95% CI	*p*
GMFCS I (*n* = 10)	Intercept	50.8	32.4 to 69.2	<.001
	Post test	18.7	9.2 to 28.2	<.001
	Intensive rehabilitation periods	1.1	−8.5 to 10.7	.801
GMFCS II (*n* = 5)	Intercept	69.4	44.4 to 94.3	.001
	Post test	6.7	3.5 to 9.9	<.001
	Intensive rehabilitation periods	2.2	−4.8 to 9.3	.373
GMFCS III (*n* = 10)	Intercept	49.7	25.0 to 74.3	.001
	Post test	9.4	5.8 to 13.0	<.001
	Intensive rehabilitation periods	2.9	−2.3 to 8.2	.225
GMFCS IV (*n* = 14)	Intercept	51.3	37.3 to 65.2	<.001
	Post test	9.9	5.5 to 14.4	<.001
	Intensive rehabilitation periods	4.4	−3.2 to 12.0	.216
GMFCS V (*n* = 7)	Intercept	49.6	31.9 to 67.3	<.001
	Post test	16.4	7.7 to 25.1	.001
	Intensive rehabilitation periods	−13.2	−63.9 to 37.4	.411

Post-test: change according to each intensive rehabilitation period, Intensive rehabilitation periods: the additive gain per period. GMFCS, gross motor function classification system. Estimates with wide CI should be interpreted with caution.

a Dependent variable.

**Figure 3:**
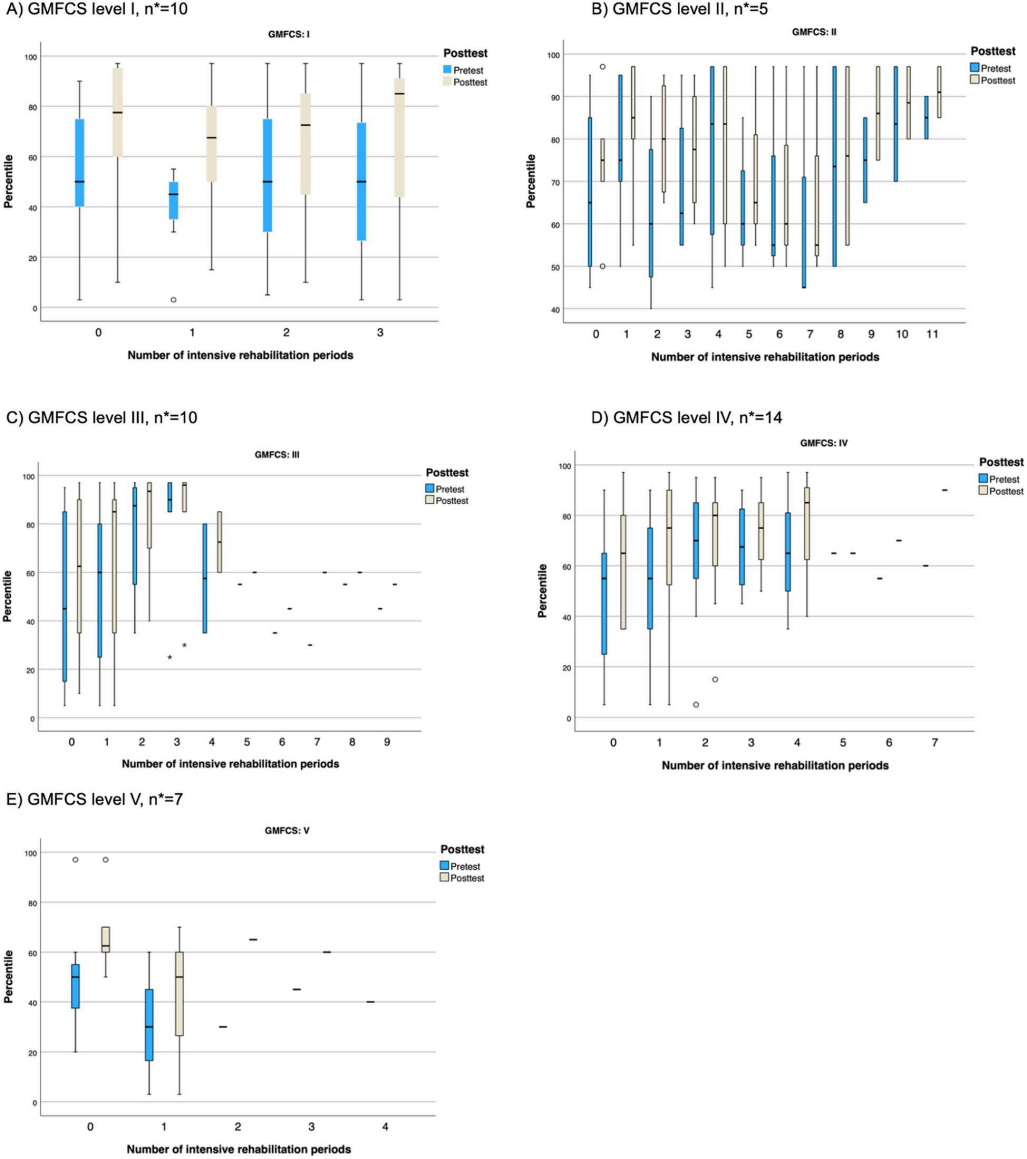
Box plots illustrating GMFM-66 percentile scores (0–100) pre- and post-test across intensive rehabilitation periods, highlighting changes over time, across the five GMFCS levels. **(A)** GMFCS level I (*n* = 10). **(B)** GMFCS level II (*n* = 5). **(C)** GMFCS level III (*n* = 10). **(D)** GMFCS level IV (*n* = 14). **(E)** GMFCS level V (*n* = 7). The black line shows the median; the box from the lower to the upper quartile is not drawn when there are too few participants. Note that no change in percentiles means progression as expected according to age and GMFCS level. A positive change in percentiles means an enhanced gross motor progress above what is expected according to age and GMFCS level. *The number of participants (*n*) at baseline.

The box plots, shown in Figure 3, suggest an increase in GMFM-66 percentiles after each intensive rehabilitation period. Most observations were in GMFCS levels II and IV, with a noticeable decrease in percentiles after intensive rehabilitation, particularly in GMFCS level II and somewhat in level IV.

**Figure 4:**
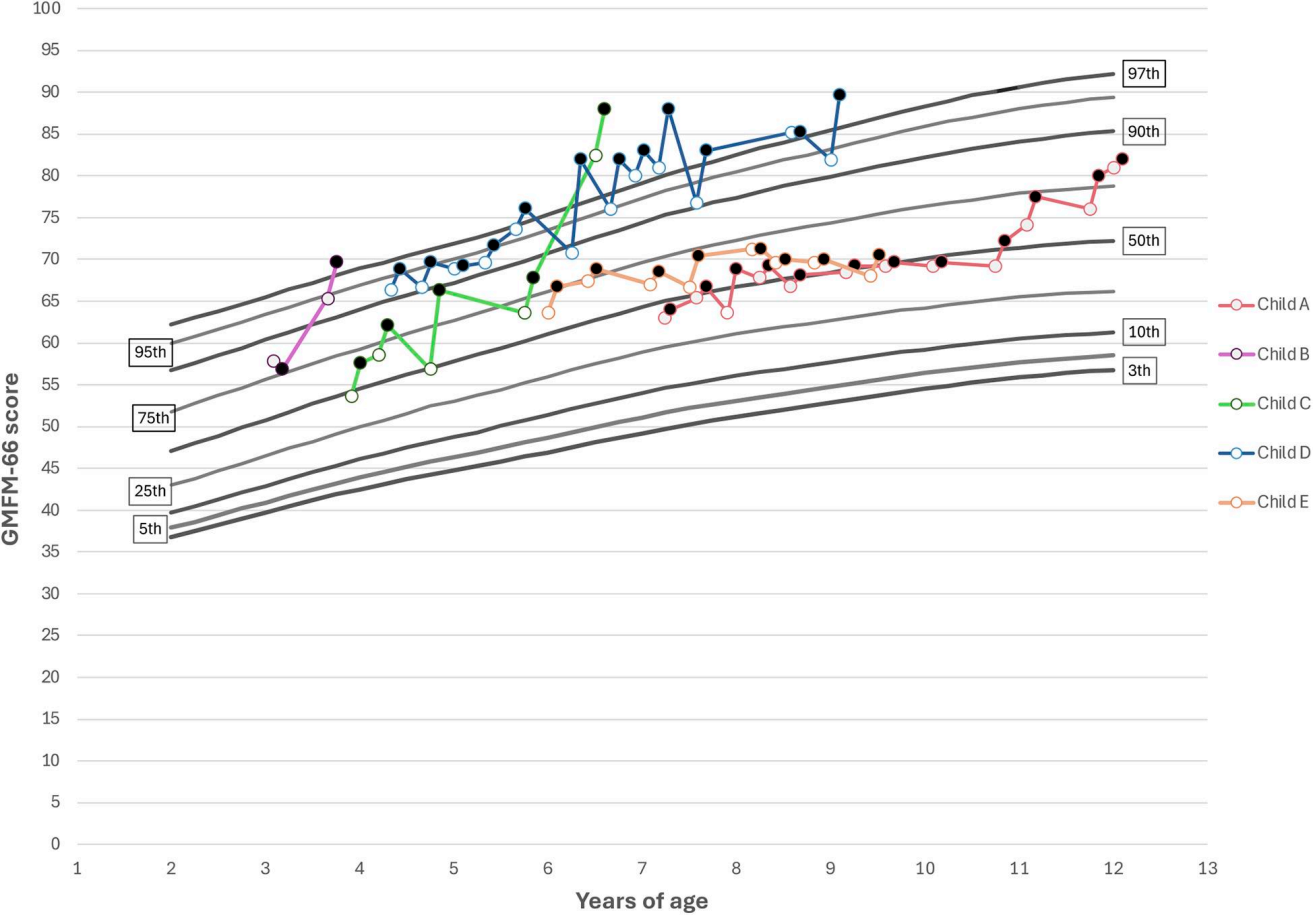
GMFM-66 pre- (open circle) and post (closed circle) scores plotted against age for children classified in GMFCS level II, shown relative to reference percentiles from Hanna et al. (28), illustrating each child's motor trajectory.

To further illustrate these findings, individual GMFM-66 scores for children in GMFCS level II were plotted against the reference percentiles from Hanna et al. (28), providing a visual representation of each child's motor function trajectory relative to expected development (Figure 4). GMFCS level II was chosen, as this subgroup included the largest number of participants who completed the most intensive rehabilitation periods, allowing for a clear visualization of longitudinal changes in gross motor function. Most children showed improvements in GMFM-66 scores over time following intensive rehabilitation periods, with gains generally aligning with or exceeding expected trajectories (28). Similarly, as seen in Figure 3, there is an observed decrease in GMFM-66 scores after each intensive rehabilitation period.

### Comparison of changes in gross motor function across age

An analysis was conducted to assess the effect of age on intensive rehabilitation effectiveness, separately for children below and above 6 years of age. Out of 46 participants, 31 were under 6 years at baseline. Both groups showed a significant increase in the GMFM-66 percentile (*p* value <0.001), with a mean change of 11.7 (95% CI: 8.6–14.9) for children under 6 years and 9.3 (95% CI: 5.8–12.9) for those over 6 years (Table 5). Additional analyses including GMFCS level or age group and their interactions with posttest and intensive rehabilitation periods revealed no significant effects.

**Table 5:** Estimated mean change (95% CI) in GMFM-66 reference percentiles^a^ based on a linear mixed model, separately for the age groups.

Age at baseline	Independent variables	Estimate	95% CI	*p*
Under 6 years	Intercept	51.7	42.0 to 61.4	<.001
	Post test	11.7	8.6 to 14.9	<.001
	Intensive rehabilitation periods	2.5	−2.4 to 7.3	.301
Over 6 years	Intercept	56.6	42.7 to 70.5	<.001
	Post test	9.3	5.8 to 12.9	<.001
	Intensive rehabilitation periods	0.8	−1.6 to 3.2	.423

Post-test: change according to each intensive rehabilitation period, Intensive rehabilitation periods: the additive gain per period.

a Dependent variable.

## Discussion

In our study, we aimed to evaluate changes in gross motor function in children with CP, aged 2–12 years, after several periods of intensive rehabilitation. These changes were assessed using the GMFM-66.

This study demonstrated that intensive rehabilitation may be associated with improvements in gross motor function in children, aged 2–12 years, with CP, across all GMFCS levels. An improvement in the average GMFM-66 scores following each period of intensive rehabilitation were noted. Children exceeded their anticipated GMFM-66 percentiles scores, with an additive gain over multiple periods. Significant changes were primarily observed during the initial periods, while a decrease in percentiles was evident between intervals of intensive rehabilitation. However, initial points for subsequent periods started, on average, at a higher level. Notably, improvements were most substantial in children classified as GMFCS levels I and V. Furthermore, the results were statistically significant for both age groups.

### Results viewed in the context of previous research

This study corroborates previous research, asserting that intensive training can enhance gross motor function in children with CP, irrespective of GMFCS levels (14, 16, 22, 26, 29, 30). Our findings underscore the potential benefits of repeated periods of intensive rehabilitation for long-term functional improvements. Observed sustained improvements in gross motor function, going beyond natural progression, align with similar studies (26, 29). Sorsdahl (29) found that at least two intensive periods were required to obtain improvements in gross motor function. Størvold's research (14) further supports that, showing an additive gain on GMFM-66 percentiles with multiple intensive periods, a finding that resonates with our results. However, the lack of statistical significance for the additive gain in our study may hint at potential confounding variables (14). This non-significant result may reflect limited statistical power due to smaller sample sizes in later periods, or a plateau effect in motor gains over time. Thus, the additive effect should be interpreted with caution.

Notably, initial periods of intensive rehabilitation may be associated with greater improvements in gross motor function than subsequent periods, an observation underpinned by prior research (26). Contrary to Sorsdahl's study (29), which reported minimal regression following two periods, we observed a regression between all intensive periods, which seemed to increase with the number of periods. The noted regression might reflect the adaptation period, where children assimilate newly acquired motor skills into everyday life. Although the program was designed to include two three-week periods over the course of a year, the actual intervals between periods varied. This variation may have impacted the consistency of the intervention and should be considered when interpreting the results.

In this study, significant improvements in GMFM-66 percentiles were particularly noted among children with GMFCS levels I and V. Prior studies have shown that children within GMFCS levels I and II, generally, exhibit more pronounced improvements in gross motor functions post-intensive rehabilitation compared to those classified in levels III–V (14, 22, 27, 29). Studies suggest that few children classified at GMFCS level V exceed their predicted percentile scores, as these individuals typically face a decline in gross motor function over time (7, 13). Nevertheless, given the small sample size in the current study's subgroup, these findings should be interpreted with caution. Individual variability, potential selection bias, or attrition of participants with decreased functionality, may have influenced the results. Additionally, the wide confidence intervals observed, particularly in GMFCS level V, further indicate uncertainty in these estimates and limit the precision with which conclusions can be drawn.

Our study found that children under the age of six have a more pronounced response to the intervention, which aligns with previous research (21, 22). The professional rationale for early intervention is based on theories of motor learning and neuroplasticity (39, 40). Children are generally expected to progress faster to their peak GMFM-score at younger ages, often plateauing around age 6–7 (28), demonstrating a stabilization of scores in GMFCS level I and II, or decline followed by a plateau in levels III–V (13). However, it's important to note that in our study significant gross motor progress was observed in both age-groups, with minimal variation. The less substantial improvements in older children may reflect their proximity to peak motor development. On the other hand, younger children might already be performing at superior levels for their age group, thereby limiting the room for further advancement. The improvement seen in the older group could also be attributed to their familiarity with organized activities, experience with functional training, and comfort in group settings (29), thus reflecting the interaction between individual maturation, task-related demands, and the broader environmental context.

### Factors contributing to enhanced motor function

Several factors are known to enhance motor function, encompassing child characteristics, alongside environmental factors and psychosocial factors, and characteristics with interventions (12, 13). The intensive rehabilitation program in this study incorporate principles know to support neuroplasticity (41). It emphasized the repeated, intensive practice of meaningful, functional activities to enhance motor, communication, social, and/or intellectual development (7). This may have contributed to the positive development in gross motor function observed in our study.

The repeated periods of intervention offered multiple learning opportunities, allowing for skills to be practiced, learned, and reinforced over time. This repeated exposure is beneficial for the consolidation and generalization of learned motor skills, with theories based on motor learning and neuroplasticity (12). The social aspect of a group setting can stimulate motivation and encourage active participation (42), in alignment with the activity and participation domains of the ICF (11). In addition, the intervention is designed to be child-centered, with goals set by both the child and their parents, further bolstering motivation and personal investment in the program.

### Strengths and limitations

This study addresses a clinically relevant question regarding the long-term development of gross motor function in children with CP. Our longitudinal study design, along with the use of repeated measurements over time, is a key strength of this study. Another strength is that the intervention is carried out at the same center with a well-established intensive rehabilitation program. Although conducted in a group setting, the program was individualized to each child's unique needs and abilities, ensuring targeted and personalized intervention strategies (31). Active parental involvement, along with targeted communication to the local physiotherapist, were seen as strengths in the program.

Another significant strength of the study lies in its utilization of the GMFM-66. The psychometric properties have been judged excellent (19, 20, 33), and the sensitivity of the GMFM-66 to detect changes in gross motor function has been well documented. We have therefore all the reason to believe that the changes observed in our study truly reflect increase in gross motor function. The assessors were familiar with the GMFM-66 assessment tool before initiation, but were, however, not blinded. Their dual role as both assessors and contributors to the intervention could introduce bias, including potential confirmation bias due to expectations about children's progress. Although the assessors were experienced and followed standardized procedures, the lack of assessor blinding remains a limitation that may affect the internal validity of the findings.

The retrospective design and lack of a control group are important limitations, both of which reduce internal validity and limit causal inference. These limitations should therefore be considered when interpreting the results. As a result, the findings suggest an association, and definitive conclusions about causality cannot be drawn. However, the use of percentiles allows for comparisons with typical developmental trajectories, which helps to mitigate the limitation of not having a control group, as it provides a recognized benchmark for progress evaluation. Although a prospective controlled design would provide stronger internal validity and allow more robust causal inferences, it was not feasible in this context because the intervention was delivered as part of an established clinical service offered to families across Norway. Implementing randomization or withholding access to the program would have posed ethical and logistical challenges.

Another limitation pertains to the lack of formal documentation on reasons for attrition, particularly evident after the fourth and fifth intervention periods. All children were initially offered two intensive rehabilitation periods, with eligibility for additional periods determined by regional health authorities. Therefore, a child may complete the two assigned periods without requiring additional ones, reflecting completion rather than dropout. Unfortunately, no data were available as to why health authorities did not assign children to further periods. Other factors may also have contributed to children not continuing participation, including family choice, logistical challenges, and medical considerations (e.g., illness, fatigue, competing priorities). A sociocultural perspective may explain why some decline further periods of intensive rehabilitation, as the intervention requires significant time (43), capacity and adaptation from both child and family. These factors may contribute to retention challenges, which should be considered when interpreting the reduced sample size in later periods and the potential for attrition bias.

While our study demonstrated improvements in GMFM-66 scores following each intensive period, the interpretation of long-term gross motor progress is more complex due to potential confounding factors. The lack of data on prior or intervening medical or surgical interventions, including selective dorsal rhizotomy (SDR), botulinum toxin and intrathecal baclofen, as well as information on socioeconomic factors (44) such as parental education and the presence of additional comorbid conditions, represents an important limitation and introduces potential confounding bias. Medical and surgical interventions may enhance motor function, subsequently leading to increased GMFM-66 scores (2, 8). Consequently, the observed improvement may not be entirely attributed to our intervention. Additionally, intellectual disability and reduced lower limb range of motion are associated with negative influences on long-term gross motor progress (14), but such information was unfortunately not available in our dataset. In addition to the structured intervention at the CPC, participants may have received varying levels of follow-up from local physiotherapists between intensive rehabilitation periods, contributing to the uncertainty of attributing observed motor function changes. Data on the amount and content of local follow-up were not available. Despite briefing local therapists on pre-established goals, the potential impact of these confounders, and our inability to control them, remains a study limitation.

### Clinical implications and future research

This study's findings highlight the significance of applying intensive treatment interventions when emphasizing gross motor progress. Future research should prioritize the implementation of standardized follow-up protocols and thorough documentation of local interventions to enhance the reliability of outcomes. Future research should aim to include prospective controlled designs, ideally with matched comparison groups, as the inclusion of a control group is essential for more robust comparisons and stronger causal inference. To corroborate our findings, additional long-term studies based on GMFM-66 percentiles, are recommended. The need for larger sample sizes is crucial to ensure precise effect size estimates, bolster statistical power, and mitigate selection bias risks, thus enhancing the generalizability of results, especially within smaller GMFCS level subgroups.

## Conclusion

This study indicates that intensive rehabilitation may be associated with improvements in gross motor function in children with CP, aged 2–12 years, spanning all GMFCS levels. Notably, improvements appeared most substantial in children classified as GMFCS levels I and V; however, the wide confidence intervals in level V, reflecting greater variability in this subgroup, warrant cautious interpretation. The results were statistically significant for all age groups, although children under 6 years at baseline showed a higher average increase. In conclusion, these findings support the potential advantages of intensive rehabilitation in enhancing gross motor outcomes in children with CP and emphasize the value of implementing repeated periods of intensive rehabilitation. This offers important insight for pediatric physiotherapists and other health care professionals working with children with CP, in optimizing treatment plans.

## Data Availability

The raw data supporting the conclusions of this article are available upon reasonable request.
